# Study of Zn-Cu Ferrite Nanoparticles for LPG Sensing

**DOI:** 10.1155/2013/790359

**Published:** 2013-06-20

**Authors:** Anuj Jain, Ravi Kant Baranwal, Ajaya Bharti, Z. Vakil, C. S. Prajapati

**Affiliations:** ^1^Applied Mechanics Department, Motilal Nehru National Institute of Technology, Allahabad 211004, India; ^2^Department of Physics, Motilal Nehru National Institute of Technology, Allahabad 211004, India

## Abstract

Nanostructured zinc-copper mixed ferrite was synthesized using sol-gel method. XRD patterns of different compositions of zinc-copper ferrite, Zn_(1−*x*)_Cu_*x*_Fe_2_O_4_ (*x* = 0.0, 0.25, 0.50, 0.75), revealed single phase inverse spinel ferrite in all the samples synthesized. With increasing copper concentration, the crystallite size was found to be increased from 28 nm to 47 nm. The surface morphology of all the samples studied by the Scanning Electron Microscopy there exhibits porous structure of particles throughout the samples. The pellets of the samples are prepared for LPG sensing characteristics. The sensing is carried out at different operating temperatures (200, 225, and 250°C) with the variation of LPG concentrations (0.2, 0.4, and 0.6 vol%). The maximum sensitivity of 55.33% is observed at 250°C operating for the 0.6 vol% LPG.

## 1. Introduction

Gas sensors are important for environmental monitoring, home safety, and chemical controlling. There is an increasing interest in the development of new materials in order to develop high performance solid-state gas sensors. Many different semiconducting oxides in bulk ceramic, thick film, and thin film forms have been studied as a sensor element for gas sensing [1, 2]. Nowadays, ferrite materials are being increasingly studied as gas sensor as their selectivity and sensitivity for some gases are comparatively better than *n*-type semiconducting oxides. Spinel-type oxide semiconductors with formula MFe_2_O_4_ have been reported to be sensitive materials to both oxidizing and reducing gases. Conduction in spinel ferrites occurs via transfer of charge carrier (electron or hole) between equal cations located in octahedral sites [3]. The main advantage of spinel-type ferrites compared to traditional metal oxide semiconductor based sensors is the ability of regulating the type of conductivity and the value of resistance by changing cation composition [4] stoichiometry [5] or annealing conditions [6]. However, information about the ferrite gas sensor in comparison with metal oxide gas sensor is still limited as reported by Sutka et al. [7]. Rezlescu et al. [8] investigated MFe_2_O_4_ (M = Cu, Cd, and Zn) as the sensing element to detect the acetone, ethanol, and LPG. Kamble and Mathe [9] reported the sensing response of nanocrystalline NiFe_2_O_4_ to various gases, namely, O_2_, LPG, ammonia, and Cl_2_. Multifunctional cubic spinel ferrite materials such as magnesium ferrite are nowadays being studied extensively for the magnetic, catalytic, biomedical, and gas sensing applications. Chen et al. [10] revealed that MgFe_2_O_4_ and CdFe_2_O_4_ were the most sensitive and selective to LPG and C_2_H_2_. Xiangfeng and Chenmou reported the gas-sensing properties of ferrites MFe_2_O_4_ (M = Zn, Cd, Mg, and Cu) thick films sensors for the sulfide sensing [11].

Gadkari et al. [12] observed that the gas sensitivity considerably depends on the ferrite composition. The mixed ferrites with varying compositions have not been studied extensively for gas sensing. Sutka et al. [7] studied the gas sensing properties of Zn-doped *p*-type nickel ferrite for acetone. Tang et al. [13] investigated the selectivity of NH_3_ by the Co_1−*x*_Ni_*x*_Fe_2_O_4_/multiwalled carbon nanotube nanocomposites. Recently nanosized ferrite materials have offered new opportunities for enhancing the properties and performances of gas sensors [14–17]. Jeseentharani et al. [18] reported the different spinel ferrite materials (MeFe_2_O_4_, M = Co, Cu, Mg, Ni, and Zn) nanoparticles for the humidity sensor. Kadu et al. [19] reported zinc-manganese Zn_1−*x*_Mn_*x*_Fe_2_O_4_ (*x* = 0.0, 0.2, and 0.4) nonmaterial for the sensing of LPG, CH_4_, CO, ethanol, and their selectivity. Darshane and Mulla [20] examined the LPG sensing phenomenon of magnesium ferrite (MgFe_2_O_4_) powder and the effect of palladium doping on operating temperature. Singh et al. [21] investigated the LPG sensing at room temperature for the nanorods and mixed shaped copper ferrite (CuFe_2_O_4_). The nickel ferrite (NiFe_2_O_4_) shows the high response for LPG sensing with respect to ethanol vapor, hydrogen sulfide, ammonia, and hydrogen at 200–450°C temperatures investigated by Darshane et al. [22]. Khandekar et al. [23] discussed the cerium (Ce) doped copper ferrite (CuFe_2_O_4_) for the gas sensing of reducing gases LPG, acetone, ethanol, and ammonia. Darshane et al. [24] reported the zinc ferrite (ZnFe_2_O_4_) nanoparticles for the LPG sensing at 250°C temperature. Banerjee et al. [25] reported the spinel ferrite Zn_1−*x*_Cu_*x*_Fe_2_O_4_ (*x* = 0.0, 0.25, 0.50, 0.75, 1.0) for the catalytic activity.

In the present study, copper and zinc mixed nanoferrite was synthesized by the sol-gel self-combustion method described in [26] and tested to sense LPG. The effect of copper concentration is studied on the sensing response of LPG as compared to pure zinc ferrite.

## 2. Experimental

### 2.1. Material Synthesis

Four samples with chemical compositions as ZnFe_2_O_4_, Zn_0.75_Cu_0.25_Fe_2_O_4_, Zn_0.50_Cu_0.50_Fe_2_O_4_, and Zn_0.25_Cu_0.75_Fe_2_O_4_ were prepared by sol-gel autocombustion method. The prepared samples have been referred to as F1, F2, F3, and F4, respectively, hereinafter in the text. The samples are prepared using following analytically pure (AR) grade Zn(NO_3_)_2_·6H_2_O (mol. wt. 287.49 gm/mol), Cu(NO_3_)_2_·3H_2_O (mol. wt. 241.60 gm/mol), and Fe(NO_3_)_3_·9H_2_O (mol. wt. 404.00 gm/mol) as starting materials. Citric acid (AR Grade, molecular weight: 210.14 g/mol) was used as chelating agent. In order to make ferrite, these reagents in desired stoichiometric ratio were mixed with citric acid in distilled water. Cold stirring was done on magnetic stirrer for 1 hour to obtain homogeneous solution. The mixture was neutralized (pH = 7) by using ammonia solution. The resulting solution was dried in an oven at 70°C for 18 hours to make the solution concentrated. Gel was obtained by constant stirring and heating the solution at 80°C temperature. The gel was subjected to calcinations at 650°C for 4.5 hours in a furnace to obtain the combusted flake form material. By grinding the flakes in mortar and pestle, powdered form of material was obtained.

### 2.2. Structural and Sensing Measurement

Structural analysis of powdered samples was carried out using X-ray diffractometer (Philips 3710, PANalytical) using Cu-K*α* radiation with wavelength (*λ* = 1.5406 Å), and the surface morphology of the samples was investigated with a Scanning Electron Microscope (JEOL-JXA-8100 SEM).

The 10 mm diameter size of pellet was made using polyvinyl alcohol (PVA) as binder of each sample for ferrite powder. Two-gram powder sample was uniformly mixed with 5 wt% by weight of PVA. The mixture was pressed in a die and punch arrangement using a hand press machine. The prepared pellets were sintered at 400°C for 2 hours to remove organic PVA. Highly conducting silver paste was applied with the help of n-butyl acetate to make the surface conductive on both sides of each pellet.

A homemade gas sensing setup was used for LPG sensing as shown elsewhere [27]. The gas-sensing characteristics were recorded with reference to time at different operating temperatures and as a function of gas concentration. The sensing response was calculated using the given formula [27]:

(1) $$\begin{array}{c} S\left(\%\right)=\left[\frac{\left(R_{a}-R_{g}\right)}{R_{a}}\right]\times 100, \end{array}$$

where *R*_*a*_ and *R*_*g*_ are the resistance in the air and in the presence of tested gas, respectively.

## 3. Results and Discussion

### 3.1. X-Ray Diffraction Analysis

**Figure 1:**
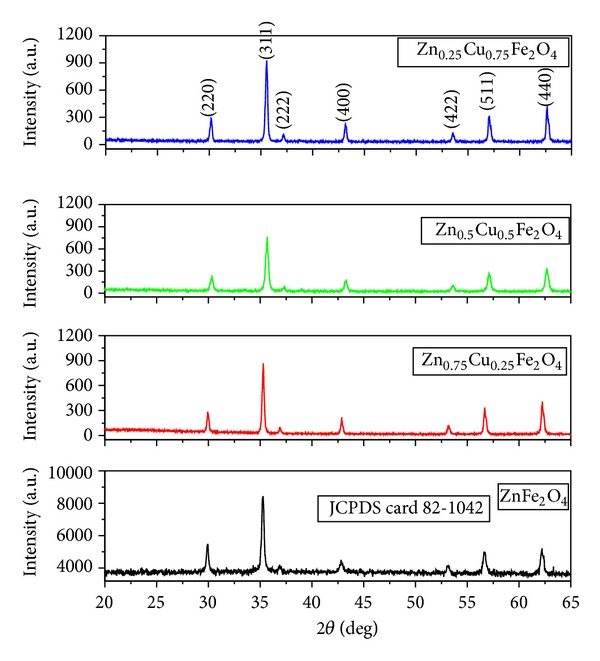
XRD spectra of the zinc-copper ferrite nanoparticles.

The observed XRD patterns of each sample have been shown in Figure 1 and found to be in good agreement with the JCPDS card 82-1042. The XRD pattern shows single peak corresponding to each diffraction angle; hence, it shows the single phase of inverse spinel zinc-copper ferrite. 

The lattice constant (*a* = *b* = *c*) has been calculated from the most prominent peak using the formula [28]

(2) $$\begin{array}{c} \frac{1}{d^{2}}=\frac{\left(h^{2}+k^{2}+l^{2}\right)}{a^{2}}\text{.} \end{array}$$

The crystallite size (*D*) and the lattice strain (*ε*) of the prepared ferrite samples have been determined using the Debye-Scherrer formula (3) [29] and the tangent formula (4) [29] as follows:

(3) $$\begin{array}{c} D=\frac{0.9\lambda }{\beta \cos\theta }\text{,} \end{array}$$

(4) $$\begin{array}{c} \varepsilon =\frac{\beta }{4\tan\theta }\text{,} \end{array}$$

where *λ* is the X-ray wavelength equal to 1.5406 Å, *θ* is the Bragg diffraction angle, and *β* (radians) is the full width at half maximum.

The lattice constant, the crystallite size, and the lattice strain of the samples thus obtained are listed in Table 1. It is observed that the crystallite size of the composite ferrites increases on increasing the Cu concentration. This is due to the decrement in the densities of nucleation centers in the doped samples which results in the formation of larger crystallite size. 

The texture coefficient has been calculated to describe the preferential orientation (*hkl*) using the following relation [30]:

(5) $$\begin{array}{c} \text{TC}\left(hkl\right)=\frac{I\left(khl\right)/I_{O}\left(khl\right)}{\left(1/N\right)\sum_{N}I\left(hkl\right)/I_{O}\left(hkl\right)}\text{,} \end{array}$$

where *N* is the number of diffraction peaks and *I*(*hkl*) and *I*_*O*_(*hkl*) are, respectively, the measured and corresponding recorded intensities according to JCPDS (082-1042) card. Texture coefficient as shown in Table 1 revealed that the crystalline nature corresponding to all the observed diffraction peaks is lower compared to the matched JCPDS card except at (422) peak. The crystallinity along this peak first increased as copper doped from F1 to F2 and then decreased to other compositions of F3 and F4 with increasing copper concentration into the zinc ferrite lattice as shown in Table 1. It is reasoned that initially copper incorporation with zinc ferrite provides the lattice energy along (422) peak growth and increases the crystallinity. As the ratio of zinc with copper is further decreased, the crystalline nature along (422) plane decreases due to increment of the substitution of zinc ion (Zn^2+^ = 0.83 Å) by copper ion (Cu^2+^ = 0.70 Å) in the zinc ferrite crystal structure. This phenomenon may be confirmed by observing the increase in the shift of the diffraction peak towards the higher angle due to the increased substitution of copper ion in place of zinc ion into the crystal lattice. This can also be explained on the basis of Vegard's law [31]. Therefore, lattice parameter also decreases with increasing copper concentration into the host lattice.

### 3.2. SEM Analysis

**Figure 2:**
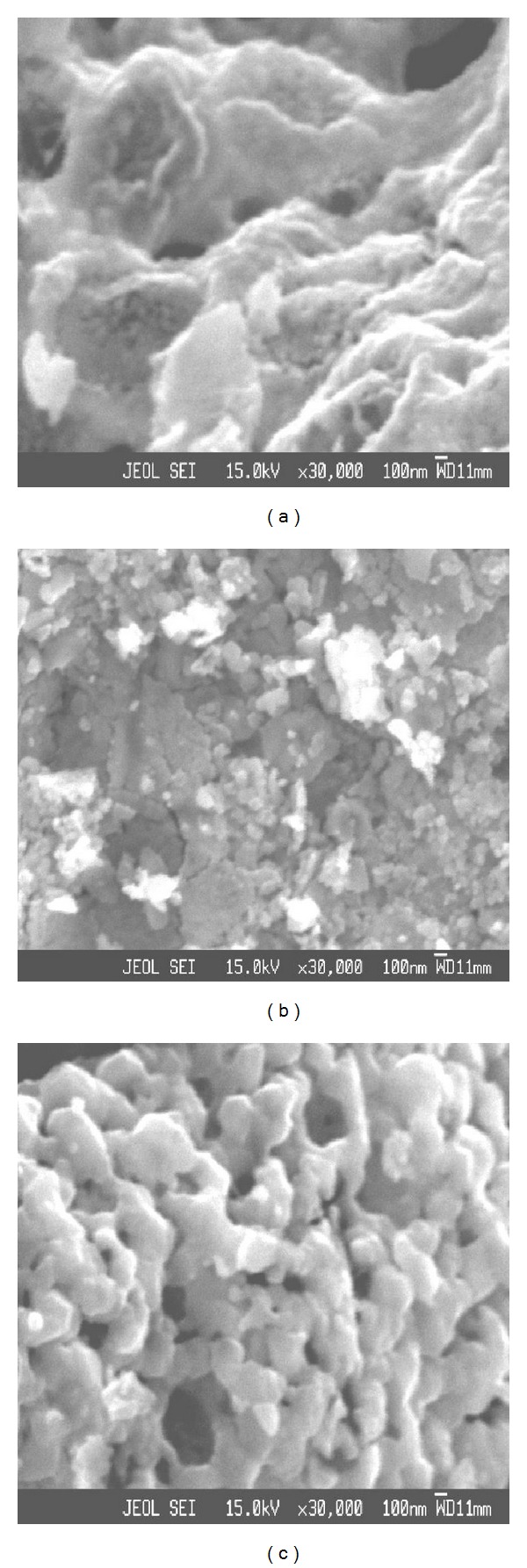
SEM micrographs of (a) Zn_1.0_Cu_0.0_Fe_2_O_4_, (b) Zn_0.75_Cu_0.25_Fe_2_O_4_, and (c) Zn_0.25_Cu_0.75_Fe_2_O_4_.

Figures 2(a)–2(c) show the SEM micrographs for all the prepared samples of ferrites. The SEM images show formation of the microagglomerated particles and also some voids. Porosity is located at the junctions of the agglomerates. It is clearly seen in the micrographs that the grains of the Zn-Cu ferrite are very rough, which allow adsorption of oxygen species on the sensing surface. Adsorption of oxygen species is responsible for gas sensing as described in Section 3.3.

### 3.3. LPG Sensing

**Figure 3:**
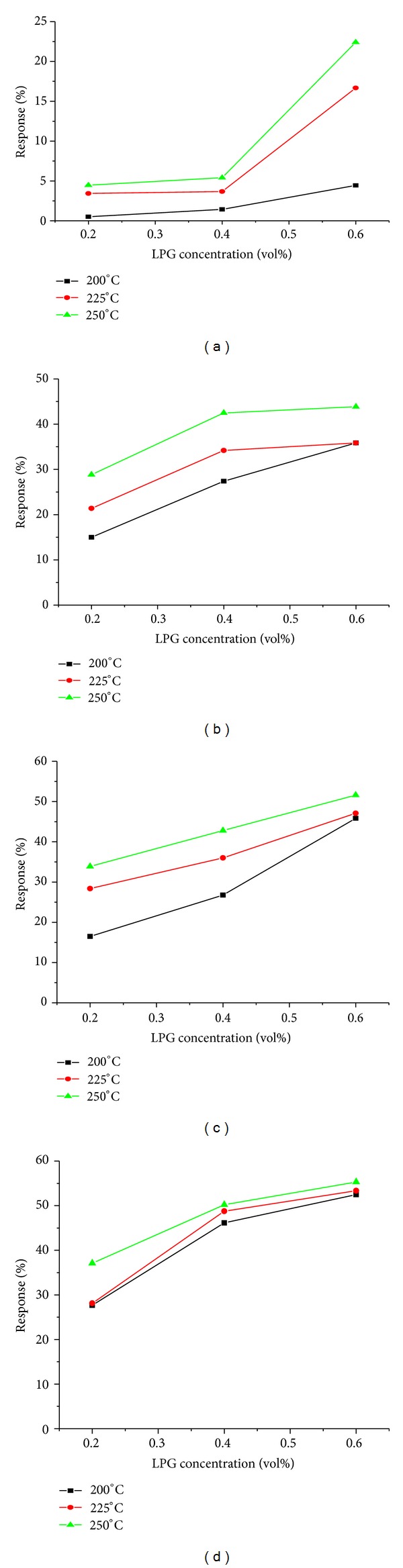
LPG response characteristics of (a) Zn_1.0_Cu_0.0_Fe_2_O_4_, (b) Zn_0.75_Cu_0.25_Fe_2_O_4_, (c) Zn_0.50_Cu_0.50_Fe_2_O_4_, and (d) Zn_0.25_Cu_0.75_Fe_2_O_4_.

The pellets fabricated as detailed in Section 2.2 were subjected in the static setup as reported in [27] for studying their sensitivity for LPG at different concentrationds of 0.2, 0.4, and 0.6 vol% at the different operating temperatures (200°C, 225°C, and 250°C). The resistance of the pellet before and after inserting the test gas was recorded. The sensitivity is then computed using (1). The results are shown in the Figure 3. As the copper concentration increased, the sensitivity increased due to more adsorption of oxygen species on the sensing sites. LPG sensing may be due to multiple microstructures such as nanoparticles, nanostructured hollow spheres, porous nature of spheres, and fine hillocks of ferrite materials dispersed uniformly on the surface. Oxygen adsorption plays an important role in the sensing properties of the pellets formed by nanosized ferrite material with multiple microstructures. Reactive oxygen species such as O_2_^−^, O^2−^, and O^−^ are adsorbed more on the surface of ferrite material at elevated temperature, and the amount of such chemisorbed oxygen ions depends strongly on operating temperature. The reaction kinetics is as follows [32, 33]:

(6) $$\begin{array}{c} {\text{O}}_{2}\;\left(\text{air}\right)⟷{\text{O}}_{2}\;\left(\text{ads}\right), \end{array}$$

(7) $$\begin{array}{c} {\text{O}}_{2}\;\left(\text{ads}\right)+{\text{e}}^{-}⟷{{\text{O}}_{2}}^{-}\;\left(\text{ads}\right), \end{array}$$

(8) $$\begin{array}{c} {{\text{O}}_{2}}^{-}\;\left(\text{ads}\right)+{\text{e}}^{-}⟷2{\text{O}}^{-}\;\left(\text{ads}\right). \end{array}$$

The oxygen species capture conduction electrons from the materials, which leads to a decrease in the electron concentration. The conversion of LPG molecule is possible at oxide surface (in presence of adsorbed oxygen ions) by dehydration as follows:

(9) CnH2n+2+2O− →H2O+CnH2n:O+e−  (dehydration).

Besides, further oxidation of the formed products is also possible; it should result in sensor response growth. Thus, the process most often proceeds through the oxidizing dehydrogenation mechanism as follows:

(10) CnH2n:O+O−   →CO2+H2O+e−  (oxidizing  dehydrogenation).

Here, C_*n*_H_2*n*+2_ represents CH_4_, C_3_H_8_, C_4_H_10_, and so forth, while C_*n*_H_2*n*_ : O represents partially oxidized intermediates on the hollow sphere surface. Thus, during oxidation LPG liberates electrons into the conduction band, thereby decreasing the resistance of the pellet upon exposure to LPG [27].

**Figure 4:**
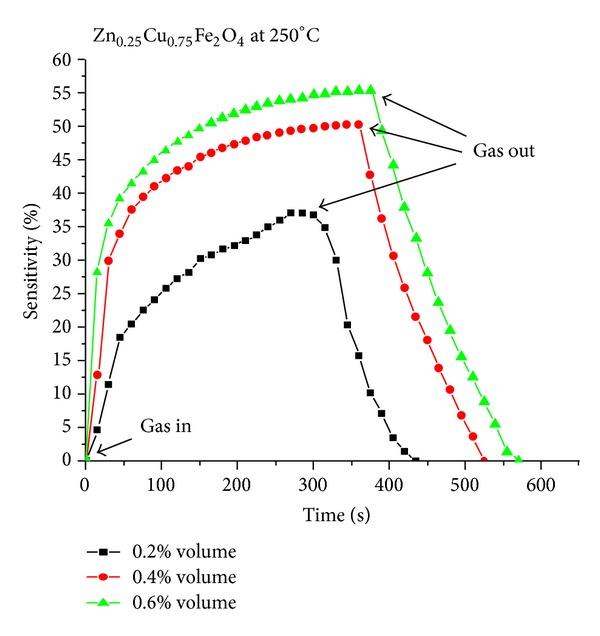
Transient response characteristics of Zn_0.25_Cu_0.75_Fe_2_O_4_ at operating temperature of 250°C.

Figure 4 shows the dynamical response of sample F4 for all the used LPG concentrations at fixed operating temperature 250°C, and it is observed that with increasing the LPG concentration, the response time and recovery time increased. This may be due to taking larger time to thermally break and react with oxygen species for higher concentration and come back to the original state for further testing. The response time recovery time of the Zn_0.25_Cu_0.75_Fe_2_O_4_ (F4) for the LPG at different concentrations 0.2, 0.4, and 0.6 vol% at operating temperatures (200°C, 225°C, and 250°C) are mention in Table 2. The maximum response found in the present study is 55.3% for 0.6 vol% LPG concentration at 250°C for F4 ferrite.

## 4. Conclusions

Mixed ferrite material Zn_(1−*x*)_Cu_*x*_Fe_2_O_4_ (*x* = 0.0, 0.25, 0.50, 0.75) was successfully synthesized by sol-gel method, and structural studies proved the nanocrystalline nature of the samples. The effect of Cu concentration and temperature on the LPG sensing and maximum sensitivity is found to be 55.33% for 0.6 vol% concentration of LPG at operating temperature 250°C for F4 ferrite. 

## Figures and Tables

**Table 1:** Structural properties of the Zn-Cu ferrites.

Zn-Cu ferrites	Positions 2*θ*(°)	*d*-spacing observed (Å)	*d*-spacing (JCPDS) (Å)	*hk* *l*			TC	Crystallite size (Å)	Lattice strain (%)	*a* = *b* = *c* (Å)	*a*-(JCPDS) (Å)
	29.915	2.9860	2.9851	2	2	0	0.4792	26.495	0.0051		
	35.158	2.5517	2.5457	3	1	1	0.5485	28.214	0.0041	8.463	8.443
	36.877	2.4366	2.4347	2	2	2	0.4687	23.086	0.0048		
ZnFe_2_O_4_	42.841	2.1102	2.1108	4	0	0	0.4900	19.289	0.0049		
	53.142	1.7229	1.7234	4	2	2	4.0714	23.237	0.0033		
	56.664	1.6239	1.6248	5	1	1	0.4830	22.743	0.0032		
	62.21	1.4918	1.4925	4	4	0	0.4561	21.695	0.0030		

	29.9547	2.9821	2.9851	2	2	0	0.3350	69.665	0.1104		
	35.3146	2.5408	2.5457	3	1	1	0.4229	47.097	0.1392	8.427	8.443
	36.9433	2.4324	2.4347	2	2	2	0.3608	60.809	0.1032		
Zn_0.75_Cu_0.25_Fe_2_O_4_	42.8465	2.1100	2.1108	4	0	0	0.4949	72.291	0.0753		
	53.21	1.7209	1.7234	4	2	2	4.3134	90.330	0.0491		
	56.6971	1.6230	1.6248	5	1	1	0.5616	53.751	0.0779		
	62.2277	1.4914	1.4925	4	4	0	0.5064	64.460	0.0597		

	30.3572	2.9435	2.9851	2	2	0	0.4177	139.581	0.0544		
	35.6702	2.5163	2.5457	3	1	1	0.5220	70.696	0.0918	8.346	8.443
	37.393	2.4042	2.4347	2	2	2	0.5294	85.270	0.0727		
Zn_0.50_Cu_0.50_Fe_2_O_4_	43.3111	2.0884	2.1108	4	0	0	0.7036	108.657	0.0496		
	53.7481	1.7049	1.7234	4	2	2	3.5573	90.5445	0.0486		
	57.102	1.6125	1.6248	5	1	1	0.4084	28.731	0.1448		
	62.6886	1.4815	1.4925	4	4	0	0.6800	78.789	0.0485		

	30.1933	2.9591	2.9851	2	2	0	0.3682	69.705	0.1095		
	35.5827	2.5223	2.5457	3	1	1	0.4296	47.132	0.1380	8.365	8.443
	37.2095	2.4156	2.4347	2	2	2	0.3699	85.225	0.0731		
Zn_0.25_Cu_0.75_Fe_2_O_4_	43.1625	2.0952	2.1108	4	0	0	0.6696	118.707	0.0455		
	53.5478	1.7108	1.7234	4	2	2	4.1639	64.599	0.0683		
	57.0276	1.6144	1.6248	5	1	1	0.4622	75.369	0.0553		
	62.6281	1.4828	1.4925	4	4	0	0.5312	55.369	0.0691		

**Table 2:** Response time and recovery time for Zn_0.25_Cu_0.75_Fe_2_O_4 _sensor.

Conc. of LPG	Temp.					
	200°C		225°C		250°C	
	Response time (min.)	Recovery time (min.)	Response time (min.)	Recovery time (min.)	Response time (min.)	Recovery time (min.)
0.2% vol	3.75	2	3.75	2.5	3.5	2.5
0.4% vol	3.75	4.25	5.5	3.5	6	3
0.6% vol	4.5	5	5.5	4	6.25	3.5
